# Nurse assistants’ experiences of encountering patients in grief due to the death of a loved one - a qualitative study in municipal health and social care

**DOI:** 10.1080/17482631.2024.2330116

**Published:** 2024-03-17

**Authors:** Anne-Lie Larsson, Ingela Beck, Ann-Christin Janlöv, Eva-Lena Einberg

**Affiliations:** a The Research Platform for Collaboration for Health, Faculty of Health Science, Kristianstad University, Kristianstad, Sweden; b The Institute for Palliative Care, Lund University and Region Skåne, Lund, Sweden

**Keywords:** A loved one, content analysis, encounter, focus group, grief, municipal health and social care, nurse assistants, patients, pay attention, qualitative study

## Abstract

**Purpose:**

The aim was to explore nurse assistants´ experiences of paying attention to and encountering patients receiving municipal health and social care, who are in grief due to the death of a loved one.

**Methods:**

A qualitative study with focus group interviews (*n* = 6) was conducted with nurse assistants (NAs) (*n* = 28) in municipal health and social care (*n* = 5) in southern Sweden. The data were analysed inductively using qualitative content analysis.

**Results:**

Three categories (1) *Noticing changes in the patient*, (2) *Using different strategies to create a dialogue with the patient*, (3) *Experiencing challenges when encountering patients in grief*, included eight sub-categories were identified. The result is captured in the theme of *Having to be attentive to signs of grief after patients´ loss of loved ones, sensing the right time to approach and comfort, while having to cope with emotional challenges.*

**Conclusions:**

The NAs encountered expressions of strong emotions from patients in grief, and even expressions of a desire to end their lives. Additionally, the NAs had to deal with their own emotions that were evoked when meeting patients in grief. These challenges indicate the need for enhanced conditions in the work culture, and improved training and supervision in health and social care.

## Introduction

Nurse assistants (NA) in municipal health and social care meet patients daily who need care due to health problems. These patients are particularly vulnerable to life-changing events (Näppä et al., 2016), such as the death of a loved one. The painful loss of a loved one is considered a part of life, and therefore the subsequent grief is a “natural” phenomenon (Hall, 2014). However, unresolved, and complicated grief is a form of mental ill health and can lead to deteriorating mental and physical health and increases the risk of death (Buckley et al., 2022; O’Connor, 2022). Furthermore, bereaved persons, often experience a lack of understanding and support from persons close to them (Jones et al., 2019). Several studies have focused on family caregivers in bereavement and grief, but few have examined the experiences of health and social care professionals when caring for bereaved patients after the death of a loved one. However, a study from Belgium (Van Humbeeck et al., 2016) has found that it can be an emotional challenge for NAs to encounter bereaved patients. Since patients in grief are at risk of further mental and physical illness, regardless of NAs’ own emotional challenges, it is important to recognize and support bereaved patients. Understanding NAs´ experiences of paying attention to and encountering patients affected by grief has the potential to enhance the care provided to patients in grief and improve their well-being.

In general, grief can be described as experienced after the loss of someone, or something significant to the person affected (Iglewicz et al., 2020). Grief initially provokes a psychological stress reaction that trigger the fight or flight response (O’Connor, 2022). It stems from emotional bonds and memories formed from the time before the death occurred (Shear, 2022). Kübler- Ross (1969) described the grieving process in terms of phases that occur in a specific order. However, Parkes (1998) states that the phases of the grieving process are not limited sequences that occur at one time. This means that persons in grief can move forward and backward between the phases, and periods of longing and despair can recur many times. Wortman and Boerner (2011) also point out that the grieving process with phases that occur in a specific order is not necessarily followed. Recent research confirms that persons grieve in different ways (Arizmendi & O’Connor, 2015).

Reviews by Buckley et al. (2022) and O’Connor (2022) show that the death of a loved one is a life event that involves suffering for the bereaved person and is associated with an increased risk of mortality and poor mental and physical health. Persons who are in grief may thus be vulnerable to additional mental illnesses in terms of psychiatric disorders, such as depression, anxiety, post-traumatic disorder, and cognitive impairment (Mason et al., 2020). Furthermore, the death of a loved one serves as a reminder of the fragility of life, which can create worry about one´s health and that of others (Carlsson et al., 2022). Patients who already have an illness or disability are particularly vulnerable to the negative impact of bereavement on their physical and mental health. NAs working in health and social care encounter patients who are in grief due to the death of a loved one as a part of their daily work.

NAs referred to in this study are those working in municipal health and social care, including positions such as nurse aides and licenced practical nurses. NAs work in municipal ordinary home care and residential care facilities (Åhlin et al., 2020, SKR, 2021). The education received by NAs is at an upper-secondary level or shorter (Statens Offentliga Utredningar [SOU], 2019). NAs provide care and social services to patients who need municipal health and social care (The Social Service Act, SFS 2001:453). These patients live in ordinary housing or residential care facilities. The residential care facilities are staffed with NAs day and night around the clock (National Board of Health and Welfare, 2016). In addition, registered nurses, who have a leading role in nursing, (including responsible for severely patients), as well as occupational therapists, assistance administrators, and physiotherapists, also work in municipal health and social care even if they do not staff the facility around the clock. If a physician is needed, the primary health care centre is contacted, usually by the registered nurse.

NAs perform personal and instrumental activities in daily life care as well as offering emotional support (The Social Service Act, SFS 2018:1724). NAs’ work also involves encountering each patient using a person-centred approach. Furthermore, NAs perform nursing tasks delegated by registered nurses (National Board of Health and Welfare, 2021a).

Patients in grief can need increased support from healthcare professionals (Hashemi et al., 2018). At the same time, Funk et al. (2017) pointed out that it could be a challenge for healthcare professionals to respond to people who are grieving. Being professional requires handling personal emotions. This can involve avoiding expressions of emotion such as crying or showing vulnerability (Van Humbeeck et al., 2016). However, the study by Van Humbeeck et al. (2016) reported that healthcare professionals experience difficulties in encountering and identifying what it means to care for patients in grief. Furthermore, they described how bereaved patients can experience feelings of loneliness and isolation, while the persons close to them may find it difficult to talk to them about the grief and understand them. An important aspect of care is that NAs pay attention to these patients. “Pay attention to” means taking notice of someone and being aware of changes in behaviour that may be associated with grief, as noted by Arizmendi and O’Connor (2015). It is thus important for NAs to be present and take an active approach to patients, showing confirmation, understanding, and care for their feelings and needs (National Board of Health and Welfare, 2001). To ultimately prevent ill health and promote well-being for patients in grief it is important to enable NAs to give support to these patients. To develop supportive interventions for NAs requires knowledge of NAs’ experiences of interacting with patients in grief. Therefore, the aim of the study was to explore nurse assistants´ experiences of paying attention to and encountering patients receiving municipal health and social care, who are in grief due to the death of a loved one.

## Materials and methods

### Design

This study has an exploratory qualitative design with focus group interviews, analysed inductively with qualitative content analysis (Graneheim & Lundman, 2004). To consider the user perspective during the research process (Bammer, 2019) a reference group was established early in the research process. The reference group consists of persons with various experiences of bereavement; a person with experience of grief related to a spouse death, a bereavement counsellor, a deacon, a registered nurse, and a nurse assistant. Both men and women are represented in the group. The reference group was involved to test the interview guide and verify that the questions were relevant and clear. Furthermore, a dialogue was held with the reference group on the preliminary results. The study followed the consolidated criteria for reporting qualitative research (COREQ) checklist (Tong et al., 2007).

### Context and setting

The study was conducted in five municipalities located in southern Sweden. The population of the municipalities ranged from 13,200 to 86,600 inhabitants. The participants worked in residential care facilities (RCF), residential care facilities for persons with cognitive impairment (RCFC) and short-term care (STC) a residential facility for persons who need temporary care for a short period of time before going home or moving to a residential care facility. These forms of care facilities are often staffed during the night and weekends solely by NAs, while the registered nurses are on call. There were also participants who worked in home care service (HCS) to provide care and service to persons in their home. Additional participants worked in home care teams (HCT) to plan and implement intensive care for patients from the hospital for a couple of weeks after the discharge. NAs in the HCT unlike the other participants worked in teams with regularly meetings every morning with registered nurse, physiotherapist and occupational therapist, and also had close contact with a physician, see Table I.

**Table I.** Overview of participants (*n* = 28) in the focus groups.

Focus group	FG 1	FG 2	FG 3	FG 4	FG 5	FG 6
Municipal Care Context	A HCT/STC	B RCFC	C RCF	C RCFC	D HCS/RCF	E HCS
Participants	3	5	4	5	7	4
Age, median (range)	49 (43–52)	49 (35–60)	44 (32–51)	53 (47–63)	54 (45–61)	48 (39–58)
Participants education addressed grief	2	0	2	2	2	0
Years in the profession, median (range)	20 (10–26)	23 (10–40)	21 (15–28)	31 (22–46)	25 (14–37)	22 (9–37)

FG = Focus group, HCS = Home Care Service, HCT = Home Care Team, RCF = Residential Care Facilities, RCFC = Residential Care Facilities for persons with Cognitive impairment, STC = Short-Term Care.

### Participants

The participants were selected through an intention known as “maximum variation sampling”. This approach aims to include a wide variety of participants in the study (Polit & Beck, 2017). The following type of selection method can provide in-depth information and understanding of the core experiences held by the participants. The inclusion criteria were NAs who work in municipal health and social care and have experience of patients in grief. The participants were recruited with help from the heads of NAs in each care unit. The intention was to use a contact person to facilitate the dissemination of written information and a video presentation of the study that was mailed to NAs in the municipalities. However, due to the heavy workload during the COVID-19 pandemic, it was not possible to use a contact person to disseminate written information and a video presentation. Instead, the NAs who wanted to participate in the study reported their interest to the head of the NAs or directly to the first author. The authors had no prior relationship with the participants.

All the NAs were women aged between 32 and 63. Of these, 27 were trained licenced practical nurses and one was a nurse aide (Table I). Five of them had a native language other than Swedish. However, they could express themselves in Swedish without any problems. Three NAs who expressed interest in participating in a focus group interview were unable to attend due to illness. Two other NAs showed up unexpectedly in two of the focus group interviews, and after informed consent, they participated instead. A total of 28 NAs participated in the study.

### Data collection

In total, six focus groups were conducted between March and June 2022, each with three to eight participants. The focus groups were homogeneous regarding occupation, but heterogeneous regarding participants’ age, years of work experience in health and social care, and if their education addressed grief. An interview guide designed for the study was pilot tested in the reference group and subsequently modified to ensure that it addressed the purpose of the study. The results of the pilot test were not included in the study. The focus group interviews were semi-structured with one researcher acting as moderator (A-L. L & I.B) and one as an observer (A-L. L, I.B & A-C. J). The focus group consisted of a moderator and an observer (Krueger & Casey, 2015). The moderator facilitated the interview using open-ended questions based on the interview guide, such as: Would you like to share your experience of encountering a patient in grief when their loved one had died?… your experience of bereaved needs?… and follow-up questions, such as: Could you say more about … could you say what you mean by … The observer role was to follow up if something needed to be clarified and to ensure that all participants had the opportunity to take part in the discussions. The focus group interviews were conducted in a separate and undisturbed room at the participants´ workplace, were audio-recorded, and took from 80 to 90 minutes.

### Analysis

Qualitative content analysis with an inductive approach was used to analyse the data (Graneheim & Lundman, 2004) with start in July year 2022. The analysis began with all authors reading the transcribed interview texts to get a sense of the whole. Thereafter, the first author read the texts several times to start the process of identifying meaning units and to note similarities and differences in content areas. As a further step, the meaning units of one transcribed focus group interview were condensed and coded by the first author. Subsequently, all authors discussed the condensation and coding of the text in order to agree on the adequacy of the process so far. After the discussion, the first author worked through and revised the meaning units, condensations and codes of this first interview text in accordance with the mutual agreement. The next step was to repeat the same process with the remaining five transcribed interview texts. Next, all the codes were sorted into “groups” based on similarities and differences in content, were labelled, and groups of codes with similar content were formed into sub-categories and thereafter into categories. On several occasions the first author (A-LL) and the last author (E-LE) discussed and adjusted codes and sub-categories as well as categories to move the analysis forward. The analysis went forward and backward. In between these activities, all authors discussed the analysis, sub-categories, and the formation of categories until consensus was reached. A preliminary result was presented to and discussed with the reference group, as a form of member check. Sub-categories and categories were established after reference group had confirmed the result and had confirmed the reasonableness of result based on their experience. The underlying meaning of the categories was then interpreted and formulated into a latent theme. The analysis was completed in September 2023 (Table II).

**Table II.** Overview of the theme, sub-categories and categories and examples of meaning units and codes.

*Theme*: Having to be attentive to signs of grief after patients´ loss of loved ones, sensing the right time to approach and comfort, while having to cope with emotional challenges			
Meaning units	Codes	Sub-categories	Categories
(NA A) … In the morning, he [the patient] just lay there. […] At night he felt that his wife was there. Physically, that is, even though she wasn’t there. And therefore, he had had a restless night./…/. (FG 1)	In the morning just lay there, had a restless night	Seeing changes in mood and behaviour	Noticing changes in the patient: The NAs described how they experienced that patients changed when affected by grief. They described how some patients changed both in mood and behaviour. These changes could vary from one moment to another. NAs also heard how the patients expressed their suffering verbally and noticed they had an increased need for help with daily life due to the loss of the loved one.
(NA A) The patient said, I feel a bit down; today would have been his [son’s] birthday or I have been to the grave today/ … /. (FG 6)	Told that they feel down. They had visited the grave of their son’s birthday	Hearing verbally expressed suffering	
(NA A) … If you lose one [the partner], you lose half of yourself. It is noticeable daily. It must be a great sorrow, and then you [the patient] may not be able to live at home anymore, when you [the patient] have lived with your husband for 60 years and everything is falling apart. (FG 1)	Life is falling apart when losing a partner and they may need to move	Noticing a changed and increased need for help	
(NA A) Sometimes you [NA] feel that it’s very difficult to explain but you can get the feeling that I should stay, or no, I should leave. Without them [the patient] having said anything. (FG 2)	Feeling to stay or leave, without the patient having said anything	Sensing when and how to approach	Using different strategies to create a dialogue with the patient: The NAs described that they used different strategies when encountering patients suffering from grief. They tried to sense when and how to approach the grieving patient depending on their needs at the time, and also tried to create a dialogue with them. Another strategy was to try to confirm the patients and establish trust in order to provide comfort.
(NA A) Trying to talk and explain, like, if it is a lady who is 94 years old/ … /so you can make them understand/ … /but you have to wrap it up [that her loved one is dead], you cannot throw it straight up, sit and talk with them. (FG 4)	Trying to talk and explain, make them understand, sit and talk with them	Trying to start a dialogue	
(NA A) Sometimes when I had time, the patient’s husband’s bed was still there so I lay down on it and we talked a bit and I read a newspaper to her [the patient], so I showed that I cared a bit. (FG 5)	I lay down next to the patient and read the newspaper to her, I showed that I cared.	Striving to confirm and establish trust	
(NA A) The most difficult [to encounter] are the ones [the patients] who get angry, if there is someone who gets really angry because yes. (FG 5)	It is difficult to encounter when patients get angry	Dealing with patient´s emotions	Experiencing challenges when encountering patients in grief: The NAs described a variety of situations that made it challenging for them to encounter patients who were grieving. Other challenging situations were dealing with their own emotions, which affected their response to patients in grief.
(NA A) … I can´t see any [patients] cry; then it starts right away. Yes, it [I start crying] starts straight away, you think to yourself about your loss as if it is still there./ … /. (FG 3)	Can not see anyone cry, thinking about own loss	Dealing with own emotions	

The authors are registered nurses with different specialist competencies, which resulted in an investigator triangulation in this study. Early in and during the research process, the authors made each other aware of their pre-understanding about grief and encountering people in grief by describing and discussing their experiences. These discussions included how to deal with pre-understanding during the research process. In addition, the moderator and the observer conducted reflections after each focus group interview, which provided a further opportunity to address the pre-understanding.

### Ethical consideration

The study received ethical approval and permission from the Swedish Ethical Review Authority (Dnr. 2021-03705). Participants received written and verbal information about the purpose of the study and what participation entailed, including that it was voluntary to participate, and they could withdraw at any time without providing an explanation. They were also informed that the interview material would be treated with confidentiality and that transcribed materials would be designated with codes. Before the interview, the participants gave written and verbal consent to participate in the study.

## Results

The overall impression of the result is captured in the theme of *Having to be attentive to signs of grief after patients´ loss of loved ones, sensing the right time to approach and comfort, while having to cope with emotional challenges*. The NAs in the study had varied experiences regarding paying attention to and encountering patients who were grieving following the death of their loved ones. The patient’s grief was not commonly discussed among the NAs, and they expressed a need to address this issue. After reflections, it became apparent that the patient’s loss and grief were not always paid attention to. The NAs worked in different care contexts (Table I), and they had similar experiences, regardless of their workplace, but certain particularly important aspects emerged. For example, regarding recognizing and encountering patients suffering from cognitive impairment, NAs needed to adapt their approach depending on whether these patients had difficulty remembering whether their loved one was alive or not, or difficulty expressing how they were feeling. Another aspect that emerged was that NAs in the HCT regularly initiated reflective conversations with the team about patients’ loss of loved ones and their grief as they often became involved when such incidents occurred. The patients’ loved ones who had died were great-grandchildren, children, spouses, siblings, and parents. The deaths that NAs referred to were both unexcepted and excepted. Unexpected death occurred through traffic accidents or suicide, and expected death was due to illness or ageing. The cases raised by NAs concerned mostly when patients’ loved ones had died unexpectedly. The results emerged as three categories and eight corresponding sub-categories (Table II).

## Noticing changes in the patient

*Seeing changes in mood and behaviour*. When the NAs encountered the patients, they were able to see noticeable shifts in their mood and behaviour following the death of a loved one. The mood changes appeared in various ways, such as patients becoming sad, tearful, or crying openly. Others became upset and angry, while some withdrew into silence or became almost apathetic. The NAs in HCT said that some patients could show anxiety, which in turn could affect their ability to sleep at night.

(NA A) … In the morning, he [the patient] just lay there. […] At night he felt that his wife was there. Physically, that is, even though she wasn’t there. And therefore, he had had a restless night./…/

(NA B) No, it was more that he was tired./ … /

(NA A) He couldn’t get up that morning because he had been awake so much./ … /

(NA C) Of Course. (FG 1)

The NAs noticed the patient’s mood could be influenced by different situations. They expressed that during the coronavirus pandemic, several patients were unable to attend the funerals of their loved ones. The NAs noticed that these patients had emotional distress due to being unable to say a final goodbye. Another situation involved patients with cognitive impairment who became sad and angry when reminded that their loved one was not alive. The NAs believed that it was as if the patient had received a death notice every time they were reminded that the loved one was not alive, and it could happen daily. They also said that when relatives of some patients directly conveyed a death message to the patient, it caused anxiety because the patient found it difficult to remember which person had passed away. Moreover, these patients’ anxiety resulted in their wandering in the corridors in search of their deceased loved ones.

The NAs noticed that some patients in grief ate less, and it was important for them to pay attention to this, as it could lead to weight loss and the risk of developing pressure ulcers. They described the changing behaviour as sometimes shifting from moment to moment. One moment the patient could be in a good mood and joking and the next moment they can be very low-key and lacking initiative. Additionally, there were some patients who increased their smoking following the death of their loved ones.

The changes in the patients’ mood or behaviour were documented in their medical records along with oral reports. This was also done when the patients´ relatives informed the NAs that one of their loved ones had died. The NAs felt the importance of sharing this information with the rest of the team, as it improved their awareness of the patients’ situation and made it easier for them to pay attention to the patients´ changed mood and behaviour.

(NA A) Then I think it’s important that staff talk, that we report all the time. Documenting deviant behaviour [of the patient]. This means that you [NA] can detect deviant behaviour or a change in the patient./ … /

(NA B) Yes, but there the important documentation./ … /

(NA C) Like, both documenting and reporting. (FG 3)

*Hearing verbally expressed suffering*. The NAs described how the patient in grief could verbally express their suffering and sometimes talked about their loss of a loved one. The patients expressed that they were feeling depressed, angry, or in a negative mood. The NAs also said that some patients expressed they had lost the will to live, had a sense of meaninglessness in life, and had the desire to die themselves. The patient could express despair and a desire to end their life, hoping to be reunited with their deceased loved one. According to the NAs, some patients who had experienced the loss of a younger loved one expressed a feeling of unfairness at being alive when their loved one was not allowed to live. Additionally, different events and situations that reminded them of the death of the loved one could be an opportunity for the patient to share their feelings.

(NA A) The patient said, I feel a bit down; today would have been his [son’s] birthday or I have been to the grave today./ … /(FG6)

The NAs found that some patients in grief often talked about memories of their loved ones and wanted to show photos and talk about what they used to do. This could happen repeatedly. At times, the patient asked for a conversation with a priest or deacon and after these conversations, the patient seemed calmer. When patients with cognitive impairment talked about their deceased loved one, it may have been because they experienced difficulties in remembering whether their loved one was alive or deceased.

*Noticing a changed and increased need for help*. The NAs described changes in the patient’s daily life, and the need for help from other people increased after the loss of a loved one. The changes varied due to the number and types of health problems. It could be that patients had depended on the deceased to handle their shared daily life. The NAs said that when the helping loved one died, the patient might have to seek extended help from others to manage their needs. This could involve receiving HCS or having to move into RCF.

(NA A) … If you lose one [the partner], you lose half of yourself. It is noticeable daily. It must be a great sorrow, and then you [the patient] may not be able to live at home anymore, where you [the patient] have lived with your husband for 60 years and everything is falling apart.

(NA B) … To realise in the midst of [grief] that you [the patient] need help for the things that the other [partner] has done…

(NA A) But if you [the patient] then lose the other half, you discover bit by bit that I [the patient] can’t cope with this, so I [the patient] must seek help for it. […] You [the patient] must be reminded all the time, this grief has to come out all the time, all the time./…/

(NA B) … Well, that’s how it is. (FG 1)

## Using different strategies to create a dialogue with the patient

### Sensing when and how to approach

The NAs explained that they need to be responsive, sensitive, and flexible in order to sense and take the opportunity to approach the patient. NAs explained that it was easier for them to approach the patient if they knew the patient’s history. Depending on who the patient was and what they thought was best for him or her, they then adapted their approach. They also tried to make eye contact and read the patient’s body language to sense their emotional state.

They also mentioned that they tried to sense when the patient wanted them to stay for a while. It also required sensitivity, and what that meant was difficult to put into words. NAs said that it was a balancing act for them to choose the moment to take the first step, such as starting a conversation about the deceased loved one. The NAs at the HCT used to ask about the patient’s interests, such as everyday activities or hobbies. They felt that when a common interest emerged, it was easier to connect with them. The NAs tried to sense when they needed to be silent or talk. They also asked questions about how the grieving patient was feeling. If the patient changed the subject, the NA took a step back and resumed the conversation at another time.

(NA A) Sometimes you [NA]feel that it’s very difficult to explain but you can get the feeling that I should stay, or no, I should leave. Without them [the patient] having said anything. (FG 2)

Furthermore, the NAs found that some patients with cognitive impairment could repeatedly ask for their loved one several times a day. In response, the NAs adapted their answers according to whether the patient was angry, sad, or happy. They could answer that their loved ones were no longer alive or try to distract the patient and talk about something else, to prevent the patient becoming even more sad or upset.

### Trying to start a dialogue

The NAs tried to create a dialogue and communicate with the patient in grief. Sometimes they even informed their colleagues in advance about their plan to talk with the patient, ensuring they would not be disturbed. If the patient started to talk to them, the NAs asked follow-up questions to encourage further discussion. If the dialogue was resumed later, they could ask the patient about what they had last talked about. The NAs acted as a sounding board, enabling a dialogue where the patient could freely express their emotions and feelings. It felt more natural to talk to the patient when they were already acquainted. However, NAs could also choose not to talk or ask about how the patient was feeling when they found out that the patient´s loved one had died. They meant that the patient had to initiate the conversation if they wanted to talk about such matters themselves. The NAs said that they did not want to intrude and impose a dialogue.

(NA A) It is very important […]to talk about grief. Then we’ve done the right thing, I think, and it makes you [the patient] feel better.

(NA B) Then it’s not something you [NA]force on them [patients]/…/.

(NA C) No, you let them [the patients] start talking in that case. You can ask how you are doing now. […] But it’s not up to me to start a conversation about a [deceased] loved one. (FG 6)

Moreover, the NAs suggested that they could try to encourage the bereaved patient to talk about the positive memories of the loved one, such as their personality traits, and share their experiences. The positive memories could be easier for the patient to discuss and provided a less sad conversation. Furthermore, the NAs said that some of the patients with cognitive impairment could ask about the deceased loved one. In response, they asked questions about their loved one, thus making the conversation more positive than sad.

(NA A) It is easier to help a person [the patient]in grief who knows that their husband has died, and he died two years ago. They may still be sad, but it is much easier to help such a person./…/

(NA B) Yes, because otherwise there will be a death notice every day.

(NA C) Yes

(NA D) Sometimes you [NA]can lead into it like, what was your [the patient] mother’s name and what was she like as a mother, then they forget it and they talk about positive memories from when they were small and their work and so on. Sometimes it goes, and they become happy again because they remember the positive things. (FG 4)

### Striving to confirm and establish trust

The NAs strove to confirm the patient’s grief and tried to create trust, to give comfort. They acknowledged that it was difficult to understand another person’s grief, but they tried to be understanding nonetheless. They told the patient that they recognized their emotional difficulties and were there for them. The NAs also described how they tried to use their body language, facial expressions, and tone of voice to make the patient feel seen and to acknowledge the patient´s grief. Sometimes they sat with the patient over a cup of coffee to show their willingness to spend time with them.

(NA A) Just sit down next to them [the patient]./…/

(NA B) Then they [the patients] become a little calmer. I think it’s nice to be able to take it easy with them.

(NA C) Mmm/…/.

(NA B) … . If we [NA and the patient] have a cup of coffee, it signals that I now have time for you [the patient]. (FG 6)

The NAs tried to establish trust with the patient by ensuring that the same NA had regular contact with the patient. When the patient self-referred to one of them, they tried to maintain that contact. They said that it was easier for them to build trust when there was a personal chemistry between themselves and the patient. NAs in HCT stated that when the patient placed their trust in them, it was easier to comfort the patient in grief, through physical closeness and touch. This could involve holding the patient’s hand, giving a hug, or placing their hand on the patient’s back. The NAs found that these gestures were reassuring to the patients. The NAs also mentioned that in situations when the patient did not want to be touched, they found that just sitting next to them and looking at photographs from the time when their loved one was alive, or reading the newspaper together, were effective to ensure that they cared for the patient.

(NA A) Sometimes when I had time, the patient’s husband’s bed was still there so I lay down on it and we talked a bit and I read a newspaper to her [the patient], so I showed that I cared a bit.

(NA B) Mmm./…/

(NA C) We can help, and we can support now, show empathy, and listen and hold hands. (FG 5)

Furthermore, when the patient with cognitive impairment felt sad, the NAs tried to distract them by playing music that the patient enjoyed while holding their hand, which seemed to calm the patient.

## Experiencing challenges when encountering patients in grief

### Dealing with patient´s emotions

The NAs described situations in which it was difficult for them to approach and create contact with the patient in grief, such as when the patient avoided eye contact and turned their head away from them. They also experienced challenges when the patient strongly acted out the grief and despair or was upset, even angry over the death of a loved one. In these situations, the patient seemed to be blocked by emotions. There were other examples where it became difficult for the NAs to approach and support the patient, such as when the patient became depressed and withdrew from them. This was evident when the NAs tried to offer them closeness or a hug.

(NA A) The most difficult [to encounter] are the ones [the patients] who get angry, if there is someone who gets really angry because yes.

(NA B) … as they [the patient] don’t want to accept it, it becomes an act of defiance.

(NA C) Mmm. (FG 5)

### Dealing with own emotions

The NAs attempted to manage their own emotions while responding to a patient in grief. They expressed feelings of sadness, and some of them found it difficult to keep from crying when they saw the patient´s sadness and tears. They also felt worried about visiting the patient, and about the difficulty of managing their own feelings in such situations. Some of them found that their thoughts and feelings reminded them of their own grief from previous losses of their loved ones. On some days it was easier for them to support the patient in grief, while on other days it felt too emotionally difficult to manage. They described the importance of adopting a professional approach to the patient in grief, assessing their ability to answer questions about emotions and listening to their answers. This could imply that the NAs sometimes tried to avoid visiting the patients in grief by asking colleagues for help.

(NA A) Yes, you [NA] are only a human.

(NA B) … You can’t be too open and vulnerable, you must know that now I have to back off, […] you have to be professional all the time, it’s a balancing act./…/

(NA C) Mmm.

(NA B) … If you have a bad day and have been too open and vulnerable, it has become too heavy that day, but thank goodness for my colleagues./…/

(NA B) Just one such thing, would you [NA] be able to take her [the patient] tomorrow? (FG 1)

## Discussion

The overall results revealed the theme of *Having to be attentive to signs of grief after patients´ loss of loved ones, sensing the right time to approach and comfort, while having to cope with emotional challenges*. The NAs generally shared similar experiences in paying attention to and encountering patients in grief regardless of workplace. However, some experiences differed, for example, the NAs working in HCT had daily team meetings involving reflective conversations with several professionals in how they could support patients in grief. When NAs in HCT found out that a patient´s loved one had died they strived to provide support by maintaining the patient’s daily routines to promote patients’ well-being and help them with grief management. Other experiences that differed were that NAs working in RCFC encountering patients with cognitive impairment and in grief were challenged when the patients would continually forget that their loved ones had died, and when they did remember, they acted as if they had received the news of the death for the first time each time it occurred. This indicates that NAs had to face different situations and seemed to be challenged to different degrees when caring for patients in grief. Regardless, NAs had to be able to manage their own emotional challenges that arose from meeting grieving patients.

The results show that NAs paid attention to patients who were severely suffering from the death of their loved ones. These patients seemed depressed and unable to take the initiative to find something to do, some were even apathetic and had a desire to die. The NAs also noticed that the patients could move from a joking mood to a depressed state. This observation aligns with Stroebe and Schut’s (1999, 2010) description of the mourners´ oscillation between being loss-oriented, with thoughts and memories occupied by the deceased, and then reacting with despair and being depressed, to being restoration-oriented, by distancing themselves from the loss and trying to adapt to living without the deceased. The results show that NAs also noticed that some patients in grief blamed themselves for being allowed to live while their loved ones did not. This self-blame sheds light on a study on bereaved persons who have experienced the loss of a younger loved one due to suicide or unexpected death. These patients felt guilt and shame, and that life was meaningless (Kõlves et al., 2020). Additionally, a report on counsellors’ experiences in working with mourners after the death of a loved one, that they could feel guilt and shame (M. Carlsson et al., 2022).

It could be confusing to distinguish between grief and depression as the signs are similar. However, these conditions differ, as grief is solely related to the death of a loved one (Shear, 2022). In addition, grief can evolve into prolonged grief disorder and is a criterion in the DSM-5 (American Psychiatric Association [APA] 2022). The results show that NAs saw that the patients in grief were depressed and they said they felt life was meaningless. Untreated prolonged grief (Buckley et al., 2022; Knowles et al., 2019; Kõlves et al., 2020), depression, loneliness, and the feeling that life is meaningless (Frumkin et al., 2021; Simon, 2013) increase the risk of suicide. In addition to suffering from mental health problems, NAs in the study noted that patients were eating less, and this could lead to physical ill health such as malnutrition. Grief can also lead to the development of other physical health problems, such as cardiovascular disease (Buckley et al., 2022). Thus, grief represents a serious threat to health, and the fact that mourners talk about having thoughts of wanting to take their own lives is alarming. Thus, professional healthcare support during the grieving process is important to prevent both the development of long-term psychological and physical ill health and to promote the well-being of the bereaved (Aoun et al., 2020). Furthermore, healthcare professionals should provide empathetic understanding and offer supportive interventions that are tailored to the unique experiences of grieving patients (McCance & McCormack, 2016).

The results showed that NAs encountered patients in grief whose loved ones had died expectedly or unexpectedly. It seemed to be easier for NAs to remember and talk about situations where the death was unexpected and dramatic. These were experiences related to patients’ in grief that NAs could have meet quite long ago. The loss had affected the patient’s life situation severely to the extent that some needed to move to a residential care facility. The patients´ losses and the changed life situation noticed by the NAs in this study, can be understood by the transition theory (Schumacher et al., 1999). According to this theory, patients in grief are in transition. A transition starts with a turning point which means moving from one stable life situation to another uncertain significant experience of a life change. Often the initial transition can trigger further transitions that the person needs to manage simultaneously. This process can have either a healthy or unhealthy impact (Schumacher et al., 1999), as shown in our study where NAs in the HCT tried to provide support to patients in grief in their everyday lives so the patient could cope with this transition. It is important for healthcare professionals to know that patients in transition are fragile and that this transition can threaten their health (Schumacher et al., 1999). According to Naylor and Keating (2008) frail persons with underlying diseases are already in a transitional phase. Therefore, NAs caring for patients in grief must be aware of what it means to be going through one or more transitional processes at the same time, as they are very vulnerable during this time and their health can be endangered.

The results bring to light that NAs met difficulties in establishing a connection with patients in grief, especially when they expressed their grief through despondency, loud crying, or were outwardly upset. There were similar difficulties when the NAs saw that bereaved needed support, such as closeness, but that the patients withdrew. Therefore, the results indicate that NAs felt inadequate in terms of how to establish connections with these patients. These challenges are in line with the study performed by Sundström et al. (2018), which finds that nursing staff felt inadequate when they were unable to support older people who shielded themselves due to existential loneliness. In the same vein, a related study on nurses’ emotional challenges in home care demonstrates that the nurses could be frustrated and felt a sense of failure when patients’ behaviour hindered them from providing necessary care and support (Eilertsen & Kiik, 2016). Some NAs found it particularly emotionally demanding when patients´ grief reminded them of their own past self-perceived grief. In these situations, they tried to distance themselves from their feelings, sometimes even avoiding meeting patients in grief. This result echoes that of Ericsson et al. (2022) report asserting that district nurses with self-experienced grief found it difficult to hold back the strong emotions that arose when they met patients in grief. Also, Rachel and Francesco (2018) concluded in their study that healthcare professionals need to alternate between distancing themselves from their own emotions, while at the same time giving support, compassion, and empathy to the bereaved. Our results show that NAs tried using different strategies to create a dialogue with patients in grief, by being sensitive and listening, making eye contact, and waiting for the right moment to ask them how they were feeling. For the NAs, deciding when and how to create the dialogue with the patient in grief involved a balancing act. The NAs in our study felt that it could sometimes be impossible for them to distance themselves from their own emotions when encountering patients in grief and they had to request support from a colleague to take care of the patient instead.

Buber’s philosophy of relationships (Buber, 1970), gives more understanding of this challenging situation, explaining that the encounter between two people naturally changes from being present and focused on an I-Thou relationship to sometimes becoming more distant and more objective, an I-It relationship (Buber, 1970). Buber´s perspective highlights the significance of NAs acknowledging the need to occasionally distance themselves and adopt an objective stance within an I-It relationship, enabling them to effectively manage their own emotions. Healthcare professionals’ own emotions should not take over their ability to provide professional care and support (Gustafsson et al., 2023). This aspect was significant for the NAs as they aimed to maintain a professional approach during meetings with patients in grief. When using a professional approach, as highlighted in Funk et al. ‘s study (2017), healthcare professionals balance between being emotionally empathic and fulfilling their professional duty to make decisions in the best interests of the patient. The NAs in our study also showed that they strived to affirm the bereaved by telling them they could see they felt bad, in order to create trust. However, according to the Danish philosopher Løgstrup and Brandby-Cöster (1994), encounters between individuals involve an ethical demand because to trust means surrendering oneself to someone else and being accepted by the other person (Løgstrup & Brandby-Cöster, 1994). In the context of professionalism, this means that NAs need to engage with the patients in grief in a way that goes beyond fulfilling formal responsibilities. Previous research reports that there is a work culture in healthcare with a focus on practical tasks such as helping patients with personal hygiene and dressing rather than a focus on relationships and emotions (Beck et al., 2012; Craftman et al., 2018). These studies thus point out that care itself is much more than solely performing practical everyday tasks. This can be linked to McCance and McCormack’s (2016) person-centred framework. To ensure a person-centred encounter, NAs need to work with the person´s beliefs and values, engage authentically, be compassionately present, and use shared decision-making when providing holistic care for a patient in grief. This will benefit the well-being and health of both parties. However, the staffing in municipal health and social care is under pressure due to a shortage of trained NAs (National Board of Health and Welfare, 2021b). Udo et al. (2018) described NAs as experiencing too many responsibilities, such as justifying various care decisions, and meeting relatives who are dissatisfied with the care. Our findings showed that, apart from HCT, NAs had no planned team meetings where they could reflect on and talk about patients´ experiences of grief and the emotions aroused in themselves and how to respond to patients in grief. They seemed to be left on their own and without real support.

To be able to provide person-centred care for patients in grief, NAs urgently need sufficient support from the health and social care organization. The support for NAs needs to be adapted to the individual and the workplace, which requires knowledge about the care environment and NAs’ individual needs. This support should promote well-functioning care staff relationships and provide NAs with the opportunity for knowledge development (McCance & McCormack, 2016). NAs need support and guidance in managing their own emotions as well as the patients´ emotions. In addition, there must be an increase in NAs’ opportunities to enhance their knowledge about grief, reflect together on existential issues, and also to have training in communication with persons in grief. This is essential to ensure that the needs for support of these patients are met and addressed. However, there is also an urgent need to investigate registered nurses’ experiences of encountering patients in grief and give NAs support in this regard.

## Methodological considerations

It is important to discuss the limitations and strengths of the study related to the concepts of trustworthiness, which are related to dependability, credibility, confirmability, and transferability (Guba, 1981). A strength of this study is the variation in age and work experience among the participants. Another strength is the site triangulation as the focus group interviews were conducted in different municipalities and care contexts. These strengths increase the credibility of the study. In terms of educational level, the focus groups were homogeneous, which may have contributed to positive interactions between NAs leading to a sense of comfort in sharing experiences with each other, which also increases the study’s credibility. Grief can be a sensitive topic to discuss as it has the potential to arouse thoughts and emotions related to one´s personal experience of grief. Consequently, the researchers strived to strengthen the trustworthiness (Guba, 1981) by being sensitive and creating a permissive environment during the focus groups, where it was acceptable for the participants to openly share their thoughts and emotions. Nevertheless, it emerged that NAs found discussing and reflecting on their experience of grief with each other rewarding since such opportunities were rare for them within their workplace.

The researchers in the current study had extensive experience of conducting focus group interviews on sensitive topics, which strengthened the study’s dependability (Guba, 1981). Another strength was that all focus group interviews were conducted by a moderator and an observer. This enabled observation of the participants’ body language and facial expressions and the interaction in the group (Krueger & Casey, 2015), which made it possible to invite participants into the discussions and follow up on ambiguities so everyone could express their experiences.

One possible limitation of the study was that all the participants were women. The NA profession is female-dominated, which could explain why no men were recruited. Regarding credibility and confirmability (Guba, 1981), all researchers discussed their respective pre-understanding throughout the research process. Furthermore, all researchers were involved in the analysis and the interpretation of the results, and we strove to restrain our pre-understanding during the analysis process. Another strength of the study was the involvement of a reference group including people with different experiences of grief. The reference group was provided with the interview guide as well as the preliminary results to solicit and discuss their feedback. The interview guide was corrected after that and the preliminary results were considered a reasonable outcome. Regarding the transferability (Guba, 1981) of the results, the NAs varied in age, work experience, education and they worked in different contexts as described in the methods section. This study was carried out in Sweden in the context of municipal health and social care. Consequently, the results could be transferred to an equivalent health and social care context; however, it is the reader who must assess the validity of such transferability.

## Conclusion

NAs encountered expressions of strong emotions from patients in grief, and even expressions of a desire to end their lives, which was difficult for the NAs to handle. Additionally, the NAs had to deal with their own emotions that were evoked when meeting patients as well as their own grief. These challenges in dealing with patients in grief indicate the need for enhanced conditions in the work culture, improved training, and supervision in health and social care.
